# Assessing the Sylvatic Yellow Fever Vectors in Southern Brazil

**DOI:** 10.3390/insects17050464

**Published:** 2026-04-30

**Authors:** Sabrina Fernandes Cardoso, Larissa Akemi Oliveira Kikuti, Andre Akira Gonzaga Yoshikawa, Iara Carolini Pinheiro, João Victor Costa Guesser, Maycon Sebastião Alberto Santos Neves, Dinair Couto-Lima, Renata Rispoli Gatti, Josiane Somariva Prophiro, André Nóbrega Pitaluga, Luísa Damazio Pitaluga Rona

**Affiliations:** 1 Departamento de Biologia Celular, Embriologia e Genética, Universidade Federal de Santa Catarina, Florianópolis 88037-000, SC, Brazil; 2 Secretaria da Saúde do Estado de Santa Catarina, Diretoria de Vigilância Epidemiológica, Florianópolis 88015-130, SC, Brazil; 3 Laboratório de Mosquitos Transmissores de Hematozoários, Instituto Oswaldo Cruz, Fundação Oswaldo Cruz, Rio de Janeiro 21040-900, RJ, Brazil; 4 Programa de Pós-Graduação em Ciências da Saúde (PPGCS), Universidade do Sul de Santa Catarina (UNISUL), Tubarão 88704-900, SC, Brazil; 5 Instituto Oswaldo Cruz, Fundação Oswaldo Cruz-Fiocruz, Rio de Janeiro 21040-900, RJ, Brazil; pitaluga@ioc.fiocruz.br; 6 Instituto Nacional de Ciência, Tecnologia e Inovação em Entomologia Molecular, Conselho Nacional de Desenvolvimento Científico e Tecnológico, Rio de Janeiro 21945-970, RJ, Brazil

**Keywords:** yellow fever, *Sabethes*, *Haemagogus*, *Aedes*, *Psorophora*

## Abstract

Yellow fever (YF) is an infectious disease caused by the yellow fever virus (YFV), an arbovirus of the *Flaviviridae* family. It is transmitted through the bite of infected mosquitoes of the Culicidae family and affects both humans and non-human primates (NHPs). This study aimed to investigate the sylvatic Culicidae fauna and the occurrence of natural YFV infection in a microregion of southern Santa Catarina, Brazil, an area recently affected by a sylvatic YF outbreak. Entomological collections were conducted between January and February 2023 in five municipalities with confirmed viral circulation. Natural YFV infection was assessed using RT-LAMP. A total of 4352 female culicids were collected, representing at least 32 species, including several key sylvatic YFV vectors. *Haemagogus leucocelaenus* was identified in all sampled municipalities, whereas *Haemagogus* (*Haemagogus*) *janthinomys* Dyar, 1921, historically considered the primary vector of sylvatic YFV in Brazil, was not detected. Mosquitoes from the genera *Aedes* Meigen, 1818; *Haemagogus* Williston, 1896; *Psorophora* Robineau-Desvoidy, 1827; and *Sabethes* Robineau-Desvoidy, 1827 were tested for YFV. Only one pool, composed of *Sabethes albiprivus*, tested positive, yielding a minimum infection rate (MIR) of 11.6. This is the first record of natural YFV infection in *Sa. albiprivus* in southern Brazil, and only the third record globally, highlighting its potential role as a secondary vector in maintaining viral circulation in sylvatic environments. Based on species presence and abundance, *Hg. leucocelaenus* is likely to have acted as the primary YFV vector in the study area. The composition of the culicid fauna, coupled with the detection of YFV in sylvatic vectors, indicates an ongoing epidemiological risk. These findings underscore the need to strengthen entomological surveillance and expand YF vaccination coverage in affected and neighbouring regions.

## Simple Summary

This study investigated sylvatic mosquito populations in a region of southern Santa Catarina, Brazil, recently affected by a yellow fever outbreak. Yellow fever is a serious mosquito-borne disease that can affect both humans and non-human primates (NHPs). Nearly 4400 mosquitoes from various species were collected, and, for the first time in southern Brazil, natural infection with yellow fever virus (YFV) was detected in *Sabethes* (*Sabethes*) *albiprivus* Theobald, 1903. This finding suggests that *Sa. albiprivus* may play a previously unrecognised role in maintaining YFV in the environment. The known vector *Haemagogus* (*Conopostegus*) *leucocelaenus* (Dyar & Shannon, 1924) was found in all sampled locations, indicating that it may have been the primary vector responsible for virus transmission in the region. These results enhance our understanding of YFV’s natural transmission cycles and provide new insights into how the virus persists in sylvatic environments. This research contributes to the fields of biology, ecology, and public health by reinforcing the importance of ongoing entomological surveillance and preventive vaccination, both of which are essential for preventing future outbreaks and protecting vulnerable populations.

## 1. Introduction

The Culicidae family comprises a large and widespread group of mosquitoes, including more than 3700 species across 113 genera, found in both temperate and tropical regions worldwide [1]. Approximately 150 of these species are known vectors of human diseases, significantly contributing to global morbidity and mortality. One such disease is yellow fever (YF), caused by the yellow fever virus (YFV) [2,3], a member of the *Flaviviridae* family and classified under the species *Orthoflavivirus flavi* [4].

Yellow fever is endemic in sub-Saharan Africa and in Central and South America, with sylvatic transmission also reported in Trinidad in the Caribbean [5], causing thousands of cases and deaths annually in Africa and South America, despite the availability of effective vaccines [6]. In Brazil, YF is classified into two main transmission cycles—sylvatic and urban—which differ significantly in vector species, vertebrate hosts, and geographic distribution [3]. No cases of the urban transmission cycle have been reported in the country since 1942 [6]. However, in 2016, the virus re-emerged, triggering the most severe sylvatic YF outbreak of the past 80 years, affecting both humans and non-human primates (NHPs) [7,8]. The virus initially spread from Venezuela into the Brazilian Amazon, then advanced through the Centre-West and Southeast regions, reaching the Atlantic Forest coastal areas and rapidly extending southward, where it remained active at least until the end of 2021 [8,9].

*Aedes* (*Stegomyia*) *aegypti* (Linnaeus, 1762) is the primary vector responsible for YFV transmission in the urban cycle, while species of the genera *Haemagogus* Williston, 1896 and *Sabethes* Robineau-Desvoidy, 1827 transmit the virus in the sylvatic cycle as primary and secondary vectors, respectively [6]. In the Americas, the most important sylvatic vector is *Haemagogus* (*Haemagogus*) *janthinomys* Dyar, 1921, which is widely distributed throughout Brazil [10]. Other *Aedes* Meigen, 1818 species, beyond *Ae. aegypti*, have also been found naturally infected with YFV [6,11]. Furthermore, mosquitoes of the genus *Psorophora* Robineau-Desvoidy, 1827 have been identified as natural hosts of the virus [3,12], indicating their potential role in transmission.

Several studies have examined potential YFV vectors in Brazil [6,13,14,15,16]. However, information on the sylvatic mosquito fauna in Santa Catarina, southern Brazil, remains scarce. This lack of data supports the hypothesis that the main species of the genera *Haemagogus* and *Sabethes*, recognised vectors of sylvatic YF in the country, were responsible for spreading the virus in the region. Therefore, this study aimed to identify sylvatic mosquito species in a microregion of southern Santa Catarina recently affected by a sylvatic YF outbreak. In addition, we assessed natural YFV infection in potential vector species to improve understanding of their role in the transmission and spread of the outbreak.

## 2. Materials and Methods

### 2.1. Study Area

**Figure 1:**
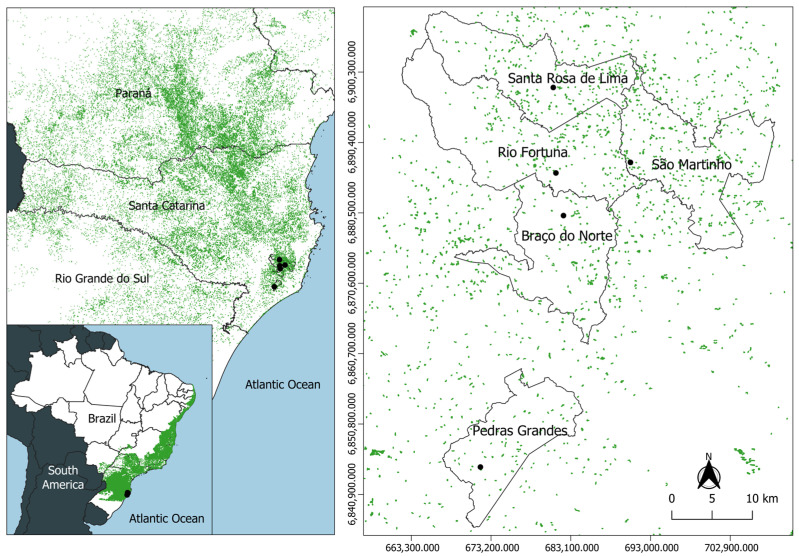
Geographic distribution of sylvatic mosquito collection sites in municipalities of southern Santa Catarina State, Brazil. The map shows, in green, the fragmented Atlantic Forest cover in Brazil and indicates the location of Santa Catarina State, along with the collection points in each municipality. The map was generated using Quantum GIS (QGIS, version 3.34) [18].

Entomological surveys were conducted in five municipalities within a microregion located in the southern part of Santa Catarina State, Brazil (Figure 1). In February 2021, YFV circulation was confirmed in the region for the first time through the detection of infected NHPs [17]. Consequently, mosquito collections were carried out in 2023 in municipalities with confirmed viral circulation: Santa Rosa de Lima (−28.032639, −49.149684), Rio Fortuna (−28.141177, −49.146304), Braço do Norte (−28.195246, −49.136690), Pedras Grandes (−28.514485, −49.242145), and São Martinho (−28.127728, −49.051721).

The five sampled areas shared similar environmental characteristics, including predominantly dense vegetation, low wind incidence, proximity to water bodies, and the presence of human dwellings along forest edges. The regional climate is classified as tropical temperate, and the remaining forest fragments belong to the Atlantic Forest biome. Historically, this ecosystem has undergone intense exploitation, resulting in largely degraded secondary forests and a highly fragmented landscape with pronounced edge effects [19].

In Santa Rosa de Lima (average altitude: 488 m), collections were conducted in the locality of Rio dos Índios. In Rio Fortuna (average altitude: 363 m), sampling took place in the Rio Otília neighbourhood. In Braço do Norte (average altitude: 238 m), collections were performed in the locality of Riacho Alegre. In São Martinho (average altitude: 353 m), samples were obtained in the Rio Gabiroba neighbourhood. Finally, in Pedras Grandes, the municipality with the lowest average altitude among the five sites (212 m), collections were conducted in the Santo Antônio neighbourhood [19].

### 2.2. Entomological Collection and Species Identification

Adult female mosquitoes were collected during the summer of 2023, specifically in January and February. Sampling was conducted between 08:00 and 17:00 on three consecutive days in each of the five municipalities. In each of the 15 collections, mosquitoes were captured simultaneously in both the tree canopy and at ground level using an entomological net and a Castro-type oral aspirator. Additionally, four CDC light traps (CDC-LT) baited with CO_2_ (dry ice) were installed in the canopy. Following collection, specimens were stored and transported on dry ice.

In the laboratory, mosquitoes were morphologically identified using a stereomicroscope (SZX16 Olympus) on a cold surface. Identification to genus and species level was carried out using standard taxonomic keys for culicids [10,20,21,22]. Non-engorged females of the genera *Haemagogus*, *Sabethes*, *Aedes*, and *Psorophora* were grouped into pools of up to 10 individuals, based on species, genus, and collection site, and stored at −80 °C for subsequent viral detection.

### 2.3. Standardisation of Viral RNA Extraction

RNA extraction was standardised based on a modified protocol described by Silva et al. [23]. To simulate viral infection, pools of up to 10 mosquitoes were homogenised in 250 µL of nuclease-free water (UltraPure™, Invitrogen, Waltham, MA, USA, cat. no. 10977015) containing 2.5 µL of RNAsecure (Invitrogen, cat. no. AM7006) and spiked with 2.5 µL of total RNA (80 ng/µL) extracted from Vero cells infected with the YFV 17D vaccine strain. After brief centrifugation, 140 µL of the supernatant was used for RNA extraction with the QIAamp Viral RNA Mini Kit (Qiagen, Hilden, Germany, cat. no. 52904), following the manufacturer’s instructions. To validate the RNA extraction, the samples were tested with RT-LAMP assays. Further details about the RT-LAMP assays can be found in the Molecular identification of YFV in mosquitoes section.

The same RNA extraction procedure was applied to mosquito pools, each containing up to 10 individuals from the genera *Haemagogus*, *Sabethes*, *Aedes*, and *Psorophora*, to detect natural YVF infection.

### 2.4. Molecular Identification of YFV in Mosquitoes

To detect YFV in captured sylvatic mosquitoes, RNA extracted from mosquito pools was used in a colourimetric RT-LAMP assay performed with the 2X WarmStart Colorimetric LAMP Master Mix kit (New England BioLabs, Ipswich, MA, USA, Protocol M1800). The assay targeted the NS1, NS5, and E genes of the viral genome, using a modified protocol based on Cardoso et al. [17], with an optimised incubation time of 60 min. Results were interpreted visually: a pink colour indicated a negative result, whereas yellow indicated a positive result. Reactions were documented with a smartphone camera. Importantly, the assay’s specificity was validated in the original study [17], which tested for cross-reactivity and found no amplification with other *Orthoflavivirus* circulating in the region.

### 2.5. Data Analysis

Quantitative and qualitative analyses of the mosquito fauna were conducted using Microsoft^®^ Excel^®^ (Microsoft 365 MSO: version 2504; Build 16.0.18730.20122). To assess species dominance and distribution, the relative abundance of each species was calculated as the proportion of individuals of a given species relative to the total number of mosquitoes collected.

Assuming that each positive pool contained only one infected mosquito, the minimum infection rate (MIR) for YFV was calculated by dividing the number of positive pools by the total number of mosquitoes of that species tested and multiplying the result by 1000 [6].

## 3. Results and Discussion

### 3.1. Abundance and Diversity of Sylvatic Culicid Fauna

This is the first study to survey the sylvatic mosquito fauna in a YF transmission area along the southern coast of Santa Catarina, Brazil. Over the study period, 15 mosquito collections were conducted across five municipalities, yielding 4352 female specimens. At least 32 taxa were identified to species level and 14 to genus level, indicating high species diversity (Table 1). The sample included genera of recognised epidemiological importance, such as *Aedes*, *Anopheles* Meigen, 1818, *Haemagogus*, and *Sabethes*, highlighting the public health relevance, as vector composition and diversity strongly influence the risk of pathogen transmission [24]. The most frequent species were *Trichoprosopon townsendi* Stone, 1944—21%, *Limatus durhamii* Theobald, 1901—9%, *Aedes* (*Ochlerotatus*) *scapularis* (Rondani, 1848)—5% and *Psorophora* (*Janthinosoma*) *ferox* (Von Humboldt, 1819)—4%.

Previous surveys in Atlantic Forest fragments of Santa Catarina reported 33 mosquito species across 14 genera, 10 of which were also detected in the present study, although with differences in composition. These discrepancies likely reflect sampling protocols: the earlier survey included late-afternoon and nocturnal collections, whereas ours was restricted to diurnal sampling. Despite this, *Ae. scapularis* was among the most abundant species in both studies, indicating consistent dominance over time [25]. Another diurnal survey in the region recorded nine species, four of which—*Ae. scapularis*, *Aedes* (*Ochlerotatus*) *serratus* (Theobald, 1901), *Ps. ferox*, and *Wyeomyia* sp. Theobald, 1901—were also found here [26]. The greater richness observed in the present study is probably due to the larger sampling effort and the use of multiple collection methods, in contrast to previous studies that relied exclusively on manual aspirators for diurnal sampling [25,26]. However, species abundance is influenced not only by the sampling method but also by the availability of nearby breeding sites. Additionally, altitudinal variation can shape microclimatic conditions, which affect mosquito population dynamics.

The mosquito abundance recorded in this study is consistent with the findings of Deus et al. [27], who also reported *Wyeomyia* and *Limatus* Theobald, 1901 among the most frequently collected genera in São Paulo State. In our data, *Wyeomyia* comprised 17% of specimens and was the most abundant genus in Pedras Grandes (284 individuals), while *Limatus* accounted for 13% and was dominant in São Martinho (462 individuals) (Table 1). These were the second and third most widespread genera observed overall.

*Trichoprosopon townsendi* was the most abundant species in our study, comprising 21% of all specimens. It was particularly common in Braço do Norte and Rio Fortuna, where 278 and 100 individuals were recorded, respectively. However, this species was not found in the survey by Deus et al. in São Paulo State [27].

In contrast, our findings are consistent with Orlandin et al. [28], who identified *Trichoprosopon pallidiventer* (Lutz, 1905) as the most abundant species in western Santa Catarina, representing over 59% of nearly 1000 specimens [29]. *Trichoprosopon* Theobald, 1901 species exploit a wide variety of natural breeding sites, particularly still water rich in organic matter and detritus. Although primarily sylvatic, they also thrive in anthropized environments, often reaching high densities in Atlantic Forest remnants [20]. This adaptability highlights their ecological resilience and ability to establish across diverse habitats [29].

Although there is no evidence of vector competence for YFV, *Trichoprosopon* mosquitoes have been linked to the transmission of several arboviruses, including Pixuna, Bussuquara, Wyeomyia, Ilhéus, and, more recently, Pirahy virus [20,30]. Further studies are needed to clarify their ecological role and vector potential.

*Aedes scapularis*, *Li. durhamii*, and *Ps. ferox* are frequently reported in high numbers in entomological surveys [6,13]. For instance, a large-scale study conducted in southeastern Brazil, covering the states of Rio de Janeiro, São Paulo, Espírito Santo, and Minas Gerais, identified *Ae. scapularis* as the most abundant species, representing 19% of more than 15,000 mosquitoes collected [6]. In the same study, *Li. durhamii* and *Ps. ferox* ranked as the sixth and the eighth most collected species among over 80 recorded taxa, further highlighting their widespread occurrence. Our findings reflect a similar pattern: *Li. durhamii*, *Ae. scapularis*, and *Ps. ferox* accounted for 9% (383 specimens), 5% (240 specimens), and 4% (187 specimens) of the total catch, respectively, making them the second, third, and fourth most abundant species in our survey.

*Aedes scapularis* displays eclectic and opportunistic host-seeking behaviour and occurs in both secondary forests and anthropogenically modified habitats [20]. The species is considered epidemiologically relevant, with at least 15 viruses isolated from field-collected specimens [31], including members of the genus *Orthoflavivirus* [20]. Experimental studies have demonstrated its competence as a vector of YFV [31]. Furthermore, *Ae. scapularis* has been implicated as a potential vector of YFV in Brazil and Colombia [31], with natural infections reported in populations from Rio de Janeiro and São Paulo [6,32].

*Limatus durhamii* is a diurnally active mosquito found in forested regions across South and Central America. It has been detected naturally infected with several arboviruses, including members of the genus *Orthobunyavirus* (family *Peribunyaviridae*) [33], as well as Zika virus (ZIKV), a member of *Orthoflavivirus* [34]. Remarkably, *Li. durhamii* is the most anthropophilic species within the tribe *Sabethini*, showing a notable ability to persist in human-modified environments [34]. The high number of specimens captured in artificial containers within Atlantic Forest fragments in São Paulo supports its adaptation to anthropized environments [33], which may also explain its presence in our samples from forest areas affected by fragmentation.

Mosquito species that have historically received limited scientific attention may nevertheless possess considerable vector potential, owing to their broad geographical ranges, adaptability to urban habitats, and anthropophilic behaviour [35]. Both *Ae*. *scapularis* and *Li*. *durhamii* exemplify this, as they are likely to participate in the transmission cycles of multiple arboviruses and should therefore be prioritised in entomological surveillance and vector competence research. Another relevant example is *Ps*. *ferox*, a species typically associated with forested habitats but also capable of feeding on humans and other vertebrates in more open environments. Field studies have documented natural infections of *Ps*. *ferox* with Rocio virus in Brazil and Venezuelan equine encephalitis virus in North America, both associated with recorded outbreaks [20,35].

Mosquitoes of the genus *Haemagogus* accounted for 2.4% of all specimens collected (103 individuals), with *Haemagogus* (*Conopostegus*) *leucocelaenus* (Dyar & Shannon, 1924), a primary sylvatic YF vector, representing 2.1% (91 individuals). This species was captured at both canopy and ground levels across all five municipalities (Table S1), but was more frequently found in the canopy, consistent with its primatophilic behaviour and known preference for feeding in the forest canopy [6,36].

The genus *Sabethes* comprised 5.1% of the total collection (222 individuals). The most frequently encountered species within this group was *Sabethes* (*Sabethes*) *albiprivus* Theobald, 1903, a recognised secondary vector of sylvatic YFV, accounting for 2% of specimens (86 individuals), and recorded in all sampled municipalities. This aligns with the findings of Abreu et al. [6], who reported *Sa*. *albiprivus* as comprising 3% of the mosquitoes collected in a similar study conducted in southeastern Brazil.

Mosquitoes of the genus *Sabethes* are diurnal, wild, and often acrodendrophilous, typically breeding in natural containers [20]. Although commonly associated with the forest canopy, they were observed across multiple vertical strata [15,22].

Arboviruses tend to attract heightened scientific attention during large-scale outbreaks or when linked to atypical or severe clinical outcomes, yet they remain insufficiently characterised under other circumstances, such as endemic circulation. Global environmental change is further accelerating the emergence and spread of these pathogens, raising concerns about novel virus-vector interactions [37]. In this context, continuous entomological surveillance and systematic monitoring are essential for early detection, risk assessment, and prevention of mosquito-borne disease outbreaks.

### 3.2. Natural Infection of Mosquitoes with Yellow Fever Virus

In Brazil, most YFV isolates from mosquitoes have been obtained from species in the *Haemagogus* and *Sabethes* genera. Nevertheless, natural infections have occasionally been reported in *Aedes* [3,13] and *Psorophora* species [3,12]. Based on this, we tested specimens from these four genera for YFV using RT-LAMP, and analysing a total of 154 pools (Table 2): 61 *Aedes*, 29 *Haemagogus*, 28 *Psorophora*, and 36 *Sabethes*.

**Figure 2:**
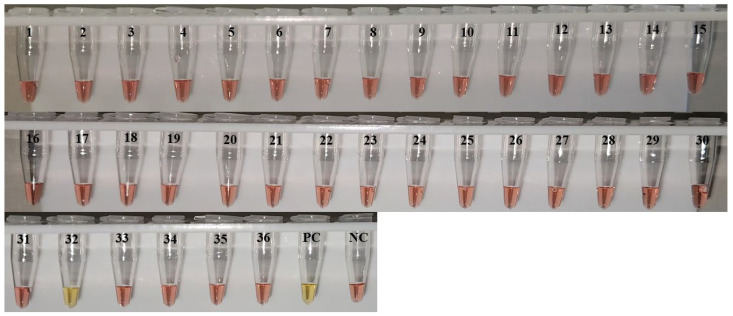
RT-LAMP analysis of mosquito pools from the genus *Sabethes*. RT-LAMP reactions were performed at 65 °C for 60 min. Yellow indicates positive results; pink indicates negative. Tubes 1–31 and 33–36 correspond to *Sabethes* pools negative for YFV. Tube 32, which comprised *Sa*. *albiprivus* specimens, tested positive for YFV. NC = negative control (nuclease-free water); PC = positive control (YFV RNA, vaccine strain 17D).

Among the 36 *Sabethes* pools, only one tested positive for YFV, pool 32, composed of *Sa. albiprivus* specimens (Figure 2). This result was confirmed by three independent retests (Figure S1). The positive pool contained 10 mosquitoes collected in the municipality of São Martinho, corresponding to a MIR of 11.63. No YFV-infected mosquitoes were detected in the remaining pools, including the 24 pools of *Hg*. *leucocelaenus* (Table 2), a species traditionally recognised as a YF vector [13].

*Sabethes albiprivus* is widely distributed across Brazilian biomes [6,15,38], with confirmed records in the southern states of Paraná [39], Santa Catarina [40], and Rio Grande do Sul [11,16]. YFV-infected individuals have also been reported in Argentina [41] and in Minas Gerais, Brazil [15]. Our study presents the first confirmed natural YFV infection in *Sa*. *albiprivus* in southern Brazil, only the second such case in the country and the third globally, highlighting its potential epidemiological importance. In the Brazilian Cerrado, particularly in northern Minas Gerais, *Sa*. *albiprivus* has been recognised as a secondary vector, with a MIR of 3.3 [15]. In our study, the recorded MIR of 11.6 is nearly three times higher, suggesting a greater role in YFV circulation in southern Brazil. Its involvement in YFV maintenance and secondary transmission has previously been proposed [6,15], and laboratory studies have confirmed its vector competence [42].

Rezende et al. [43] reported the introduction and permanence of YFV in southeastern Brazil from 2016 to 2018, emphasising that ecological and climatic conditions outside the Amazon Basin, such as those in the Atlantic Forest, can support viral maintenance during interepidemic periods. Our findings align with this, as YFV was detected in 2023, nearly two years after the 2021 sylvatic outbreak, indicating continued circulation despite the absence of active transmission. This persistence likely reflects complex vector-host dynamics and possible hidden transmission cycles that may remain undetected. Overall, the evidence suggests that *Sa*. *albiprivus* played a secondary role in YFV transmission and contributed to viral maintenance in the region.

During the 2021 sylvatic YFV outbreak in southern Santa Catarina, NHPs of the genus *Alouatta* Lacépède, 1799, acted as the main amplifying hosts of YFV and suffered high mortality. Although NHPs are central to the sylvatic transmission cycle, susceptibility differs across species, with some surviving infection and potentially acting as transient reservoirs [44]. The detection of neutralising antibodies in other mammals, including agoutis, porcupines, and anteaters [45], further suggests that these species may act as incidental hosts, potentially expanding the range of mosquito vectors involved.

Although *Sa*. *albiprivus* is typically associated with the forest canopy, these mosquitoes also occur across multiple vertical strata, which increases their likelihood of contact with diverse vertebrate hosts [15,22] and thereby facilitates YFV transmission. The ability of YFV to infect several mammalian species and to be transmitted by multiple mosquito taxa likely contributes to its persistence in natural environments [46]. Vertical transmission in mosquitoes may also help maintain the virus during interepidemic periods. However, further research is needed to clarify the role of non-primate mammals and vertical transmission in sustaining the sylvatic YFV cycle. Evidence also suggests that YFV can persist in the Atlantic Forest for at least three consecutive transmission seasons without requiring new introductions [47].

During the last sylvatic YF outbreak in Brazil, *Hg*. *leucocelaenus* was considered a primary vector due to its wide distribution, abundance, and high natural infection rates [6]. This species has been detected with YFV in several regions [6,44], including the southern state of Rio Grande do Sul [16]. In our study, 103 *Haemagogus* specimens were collected, including 91 *Hg*. *leucocelaenus*, yet none tested positive for viral RNA, despite their presence across all five sampled municipalities affected by the outbreak in Santa Catarina.

The absence of viral detection in *Hg*. *leucocelaenus* may reflect low viral circulation at the time of sampling in the absence of the main amplifying hosts (*Alouatta* spp.), and low viral loads in mosquitoes. In addition, dilution of genetic material during pool formation, which can reduce sensitivity, especially in pools with low viral loads, may have underestimated the YFV infection rate. Furthermore, the MIR calculation assumes only one infected mosquito per positive pool; therefore, for negative pools, the true infection rate may be higher. Thus, a reported MIR of zero represents only a lower-bound estimate.

The RT-LAMP assay, however, proved to be a reliable tool for detecting YFV in mosquitoes, having been previously optimised through reaction adjustments, validation with varying viral concentrations, and rigorous cross-contamination control [17]. Although the 153 pools tested negative, the collections yielded valuable insights into mosquito activity and dispersal in the region and confirmed residual viral circulation in the secondary vector *Sa*. *albiprivus*.

Although *Hg*. *janthinomys* is traditionally recognised as the primary sylvatic vector of YFV in Brazil [6,13,20], with a distribution ranging from northern Argentina and southern Brazil to Honduras and Nicaragua [20], both our study and previous surveys [48] reported a pseudo-absence of this species in Santa Catarina. This is noteworthy because Li et al. [48] modelled its environmental suitability and identified Santa Catarina as a highly suitable area for its occurrence. Despite using effective collection methods, including entomological nets and canopy traps baited with CO_2_ [27], we did not detect *Hg. janthinomys* in any of our sampling sites. In contrast, *Hg. leucocelaenus* was consistently collected across the study area. The absence of *Hg. janthinomys* therefore suggests that *Hg. leucocelaenus*, together with *Sa. albiprivus*, may have played a key role in sustaining transmission during the recent YF outbreak in Santa Catarina. *Haemagogus leucocelaenus* accounted for 2.1% of the total mosquitoes and was detected in all municipalities, comparable in abundance and distribution to *Sa. albiprivus*, the only species found YFV-positive in this study. Although infection was observed in *Sa. albiprivus*, the widespread presence and well-documented high vector competence of *Hg. leucocelaenus* strongly support its role as the primary vector in the region.

The five municipalities surveyed are small, with urban areas directly bordering natural habitats. These regions are characterised by fragmented vegetation linked by ecological corridors, facilitating mosquito movement in search of blood meals [8,20]. The high dispersal capacity of YFV vectors, even in partially deforested landscapes [20], combined with declines in NHP populations due to habitat loss and YF-related mortality, reduces available blood sources and promotes mosquito dispersal [8,14]. This highlights the need for continuous and integrated surveillance capable of detecting silent transmission, even in areas where human cases or epizootics are absent. Under these conditions, the surveyed municipalities represent high-risk areas for YFV transmission. Consequently, widespread yellow fever vaccination campaigns are strongly recommended in these and surrounding regions, given the ongoing circulation of YFV and its sylvatic vectors.

## 4. Conclusions

This study represents the first survey of sylvatic mosquito fauna with natural YFV infection in the state of Santa Catarina. The detection of naturally infected *Sa*. *albiprivus* highlights its role as an important vector in the maintenance and transmission of the virus.

The absence of *Hg*. *janthinomys* and the presence of *Hg*. *leucocelaenus* suggest a potential change in local vector dynamics, particularly in areas where NHP populations were severely impacted.

Given the continued circulation of the virus and the presence of competent vectors, this study underscores the importance of sustained entomological surveillance and expanded vaccination coverage to prevent future outbreaks in the region.

## Figures and Tables

**Table 1:** Adult mosquito species, grouped by genus, collected from January to February 2023 in five municipalities of Santa Catarina State, southern Brazil.

Mosquito Species	Total	Relative Abundance %	Collection Municipalities ^a^				
			BN	PG	RF	SM	SR
*Aedes argyrothorax*	2	0.05	2				
*Aedes crinifer*	20	0.46			1		19
*Aedes fluviatilis*	6	0.14	5			1	
*Aedes fulvithorax*	1	0.02			1		
*Aedes rhyacophilus*	5	0.11		2	3		
*Aedes scapularis*	240	5.51	57	128	32	4	19
*Aedes serratus*	37	0.85		3	8	21	5
*Aedes terrens*	41	0.94	25	7		9	
*Aedes* sp.	133	3.06	26	8	41	30	28
*Anopheles cruzii*	168	3.86	2	114	17	28	7
*Anopheles eiseni*	1	0.02			1		
*Anopheles lutzi*	5	0.11				1	4
*Anopheles maculipes*	2	0.05					2
*Anopheles neivai*	1	0.02			1		
*Anopheles* sp.	62	1.42	4	39	3	11	5
*Coquillettidia* *venezuelensis*	1	0.02					1
*Coquillettidia* *shannoni*	2	0.05			1		1
*Coquillettidia* sp.	1	0.02					1
*Culex* sp.	54	1.24	29	4	12	3	6
*Haemagogus* *leucocelaenus*	91	2.09	44	8	8	25	6
*Haemagogus* sp.	12	0.28	8	2	1	1	
*Johnbelkinia* sp.	4	0.09	3				1
*Limatus* *durhamii*	383	8.80	83	143	60	92	5
*Limatus* *paraensis*	48	1.10	3	6	19	20	
*Limatus* sp.	571	13.12	43	21	45	462	
*Mansonia wilsoni*	2	0.05			1		1
*Mansonia* sp.	29	0.67			29		
*Psorophora* *ferox*	187	4.30	8	4	94	27	54
*Psorophora* *lutzii/amazonica*	2	0.05				2	
*Psorophora* sp.	29	0.67			11	12	6
*Runchomyia* sp.	99	2.27	7	72	5	11	4
*Sabethes* *albiprivus*	86	1.98	25	16	5	10	30
*Sabethes* *aurescens*	1	0.02					1
*Sabethes* *idiogenes*	21	0.48	18				3
*Sabethes* *intermedius*	60	1.38	30	6		2	22
*Sabethes* *melanonymphe*	28	0.64	14		1	1	12
*Sabethes* *purpureus*	3	0.07	1			1	1
*Sabethes* *xhyphydes*	8	0.18	5	2			1
*Sabethes* sp.	15	0.34	7			1	7
*Shannoniana* sp.	9	0.21	1	3			5
*Trichoprosopon* *digitatum*	71	1.63	11	11	7	37	5
*Trichoprosopon* *townsendi*	904	20.77	278	131	100	375	20
*Trichoprosopon* *soaresi*	12	0.28		5		2	5
*Trichoprosopon* *vonplesseni*	43	0.99	5		14		24
*Trichoprosopon* sp.	105	2.41	15			83	7
*Wyeomyia* sp.	747	17.16	166	284	64	183	50
**Nº mosquito total**	**4352**	**100**	**925**	**1019**	**585**	**1455**	**368**

^a^ Collection municipalities: BN: Braço do Norte; PG: Pedras Grandes; RF: Rio Fortuna; SM: São Martinho; SR: Santa Rosa de Lima.

**Table 2:** Numbers of adult mosquitoes from the genera *Sabethes*, *Haemagogus*, *Aedes*, and *Psorophora* tested for YFV infection, grouped by the number of pools tested, showing the number of positive pools and the corresponding minimum infection rates (MIR).

Mosquito Species	Nº Mosquitoes	Pools Tested (Positives)	MIR
*Sabethes* *albiprivus*	86	10 (1)	11.63
*Sabethes* *intermedius*	60	8 (0)	0
*Sabethes* *melanonymphe*	27	5 (0)	0
*Sabethes* *idiogenes*	21	3 (0)	0
*Sabethes* *xhyphydes*	8	3 (0)	0
*Sabethes* *purpureus*	3	3 (0)	0
*Sabethes* *aurescens*	1	1 (0)	0
*Sabethes* sp.	15	3 (0)	0
*Haemagogus* *leucocelaenus*	91	24 (0)	0
*Haemagogus* sp.	12	5 (0)	0
*Aedes argyrothorax*	2	1 (0)	0
*Aedes crinifer*	20	3 (0)	0
*Aedes fluviatilis*	6	2 (0)	0
*Aedes fulvithorax*	1	1 (0)	0
*Aedes rhyacophilus*	5	2 (0)	0
*Aedes scapularis*	240	26 (0)	0
*Aedes serratus*	37	6 (0)	0
*Aedes terrens*	41	5 (0)	0
*Aedes* sp.	133	15 (0)	0
*Psorophora* *ferox*	187	21 (0)	0
*Psorophora* *lutzii/amazonica*	2	1 (0)	0
*Psorophora* sp.	29	6 (0)	0
**Total**	**1027**	**154**	**11.63**

## Data Availability

The datasets used and/or analysed the current study are available from the corresponding author upon reasonable request.

## Supplementary Materials

The following supporting information can be downloaded at: https://www.mdpi.com/article/10.3390/insects17050464/s1, Table S1: Mosquito species captured by municipality. Collection sites: Braço do Norte (BN), Pedras Grandes (PG), Rio Fortuna (RF), São Martinho (SM), Santa Rosa de Lima (SRL). Canopy-level collection: Specimens were captured using CDC-type traps baited with CO_2_ (CDC), as well as manually using an entomological net and a Castro-type oral aspirator (NET). Ground-level collection: Mosquitoes were manually collected at ground level using an entomological net and a Castro-type oral aspirator.; Figure S1: RT-LAMP assay performed on a *Sabethes albiprivus* positive pool for YFV. The reaction was performed in triplicate. Reaction temperature: 65 °C; reaction time: 60 min. Yellow indicates a positive result; pink indicates a negative result. R1–R3: replicates 1 to 3. NC: negative control (nuclease-free water); PC: positive control (YFV RNA, vaccine strain 17D).

## Abbreviations

The following abbreviations are used in this manuscript:

NHP	Non-human primates
YFV	Yellow fever virus
YF	Yellow fever
M	Metres
MIR	Minimum infection rate
RT	Reverse transcription
LAMP	Loop-mediated isothermal amplification
CDC-LT	CDC Light traps
RNA	Ribonucleic acid
ZIKV	Zika virus
NC	Negative control
PC	Positive control
