# Supplementary material for: Assessing the Sylvatic Yellow Fever Vectors in Southern Brazil
Source: Insects. 2026 Apr 30;17(5):464. doi: 10.3390/insects17050464 (PMC13207325; doi:10.3390/insects17050464)

SUPPLEMENTARY INFORMATION

Table S1. Mosquito species captured by municipality. Collection sites: Braço do Norte (BN), Pedras Grandes (PG), Rio Fortuna (RF), São Martinho (SM), Santa Rosa de Lima (SRL). Canopy-level collection: Specimens were captured using CDC-type traps baited with CO_2 (CDC), as well as manually using an entomological net and a Castro-type oral aspirator (NET). Ground-level collection: Mosquitoes were manually collected at ground level using an entomological net and a Castro-type oral aspirator.

Mosquito species	BN			PG			RF			SM			SRL			Total
	Canopy		Ground	Canopy		Ground	Canopy		Ground	Canopy		Ground	Canopy		Ground	
	CDC	NET		CDC	NET		CDC	NET		CDC	NET		CDC	NET		
Aedes argyrothorax		2														2
Aedes crinifer								1						3	16	20
Aedes fluviatilis		1	4								1					6
Aedes fulvithorax							1									1
Aedes rhyacophilus				1	1			2	1							5
Aedes scapularis	1	25	31	4	64	60	1	17	14		1	3	7	4	8	240
Aedes serratus					3		1	4	3	5	8	8		1	4	37
Aedes terrens	1	12	12		5	2				2	2	5				41
Aedes sp.	2	8	16	1	3	4		6	35	9	7	14	2	2	24	133
Anopheles cruzii		2		21	39	54		14	3	2	20	6	2	1	4	168
Anopheles eiseni								1								1
Anopheles lutzi											1				4	5
Anopheles maculipes													1	1		2
Anopheles neivai									1							1
Anopheles sp.		4		1	28	10		3		1	7	3	1	4		62
Coquillettidia venezuelensis															1	1
Coquillettidia shannoni								1						1		2
Coquillettidia sp.														1		1
Culex sp.	8	8	13		2	2	5	5	2	1	2			1	5	54
Haemagogus leucocelaenus	6	25	13		7	1	2	5	1	3	13	9		5	1	91
Haemagogus sp.	1	3	4		1	1		1			1					12
Johnbelkinia sp.			3										1			4
Limatus durhamii	18	24	41	21	42	80	4	24	32	19	37	36	2	1	2	383
Limatus paraensis	2	1		1	3	2	4	7	8	13	4	3				48
Limatus sp.	11	14	18	6	4	11	7	11	27	156	110	196				571
Mansonia wilsoni							1								1	2
Mansonia sp.							5	7	17							29
Psorophora ferox	1	3	4		1	3	9	33	52	10	5	12	5	16	33	187
Psorophora lutzii/amazonica										2						2
Psorophora sp.							4	2	5		6	6		2	4	29
Runchomyia sp.	2	4	1	14	20	38	1	2	2	4	5	2	3		1	99
Sabethes albiprivus	13	9	3	4	10	2	3		2	4	4	2	9	18	3	86
Sabethes aurescens															1	1
Sabethes idiogenes	5	7	6												3	21
Sabethes intermedius	4	18	8		4	2				1	1		2	17	3	60
Sabethes melanonymphe	3	8	3					1			1		1	8	3	28
Sabethes purpureus		1										1		1		3
Sabethes xhyphydes		5			2									1		8
Sabethes sp.	3	2	2							1			2	2	3	15
Shannoniana sp.			1	1	2									2	3	9
Trichoprosopon digitatum	2		9	1	5	5		3	4	1	13	23		2	3	71
Trichoprosopon townsendi	42	56	180	15	57	59	21	33	46	95	64	216		0	20	904
Trichoprosopon soaresi					4	1					1	1		2	3	12
Trichoprosopon vonplesseni	2		3				3	7	4				5	4	15	43
Trichoprosopon sp.	2	3	10							54	2	27	3	1	3	105
Wyeomyia sp.	20	87	59	51	141	92	30	15	19	77	72	34	17	23	10	747
**Nº mosquito total**	**149**	**332**	**444**	**142**	**448**	**429**	**102**	**205**	**278**	**460**	**388**	**607**	**63**	**124**	**181**	**4352**

Figure S1. RT-LAMP assay performed on a *Sabethes albiprivus* positive pool for YFV. The reaction was performed in triplicate. Reaction temperature: 65 °C; reaction time: 60 minutes. Yellow indicates a positive result; pink indicates a negative result. R1–R3: replicates 1 to 3. NC: negative control (nuclease-free water); PC: positive control (YFV RNA, vaccine strain 17D).

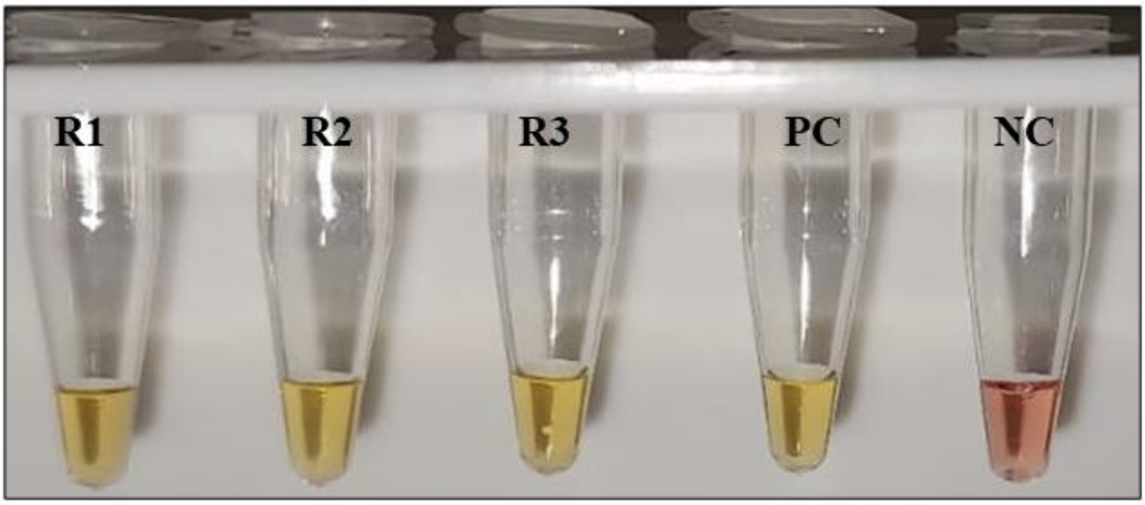

Supplement: Supplementary file 1 [file insects-17-00464-s001.zip › insects-4002453-supplementary.pdf]
